# Evaluating the impact of Mexico’s drug policy reforms on people who inject drugs in Tijuana, B.C., Mexico, and San Diego, CA, United States: a binational mixed methods research agenda

**DOI:** 10.1186/1477-7517-11-4

**Published:** 2014-02-12

**Authors:** Angela M Robertson, Richard S Garfein, Karla D Wagner, Sanjay R Mehta, Carlos Magis-Rodriguez, Jazmine Cuevas-Mota, Patricia Gonzalez Moreno-Zuniga, Steffanie A Strathdee

**Affiliations:** 1 Department of Epidemiology, Harvard School of Public Health, 677 Huntington Avenue Kresge Building, Room 911, Boston, MA 02115, USA; 2 The Fenway Institute, Fenway Health, 1340 Boylston St, 8th floor, Boston, MA 02215, USA; 3 Division of Global Public Health, School of Medicine, University of California at San Diego, 9500 Gilman Drive, La Jolla, CA 92093-0507, USA; 4 Division of Infectious Diseases, School of Medicine, University of California at San Diego, 9500 Gilman Drive, La Jolla, CA 92093-0711, USA; 5 Centro Nacional para la Prevención y Control del VIH y el SIDA, Secretaría de Salud de México, México

**Keywords:** Injection drug use, HIV, Hepatitis C virus, M. tuberculosis, Drug policy reform, Structural interventions, Decriminalization, Mixed methods, International collaboration, Mexico

## Abstract

**Background:**

Policymakers and researchers seek answers to how liberalized drug policies affect people who inject drugs (PWID). In response to concerns about the failing “war on drugs,” Mexico recently implemented drug policy reforms that partially decriminalized possession of small amounts of drugs for personal use while promoting drug treatment. Recognizing important epidemiologic, policy, and socioeconomic differences between the United States—where possession of any psychoactive drugs without a prescription remains illegal—and Mexico—where possession of small quantities for personal use was partially decriminalized, we sought to assess changes over time in knowledge, attitudes, behaviors, and infectious disease profiles among PWID in the adjacent border cities of San Diego, CA, USA, and Tijuana, Baja California, Mexico.

**Methods:**

Based on extensive binational experience and collaboration, from 2012–2014 we initiated two parallel, prospective, mixed methods studies: *Proyecto El Cuete IV* in Tijuana (n = 785) and the STAHR II Study in San Diego (n = 575). Methods for sampling, recruitment, and data collection were designed to be compatible in both studies. All participants completed quantitative behavioral and geographic assessments and serological testing (HIV in both studies; hepatitis C virus and tuberculosis in STAHR II) at baseline and four semi-annual follow-up visits. Between follow-up assessment visits, subsets of participants completed qualitative interviews to explore contextual factors relating to study aims and other emergent phenomena. Planned analyses include descriptive and inferential statistics for quantitative data, content analysis and other mixed-methods approaches for qualitative data, and phylogenetic analysis of HIV-positive samples to understand cross-border transmission dynamics.

**Results:**

Investigators and research staff shared preliminary findings across studies to provide feedback on instruments and insights regarding local phenomena. As a result, recruitment and data collection procedures have been implemented successfully, demonstrating the importance of binational collaboration in evaluating the impact of structural-level drug policy reforms on the behaviors, health, and wellbeing of PWID across an international border.

**Conclusions:**

Our prospective, mixed methods approach allows each study to be responsive to emerging phenomena within local contexts while regular collaboration promotes sharing insights across studies. The strengths and limitations of this approach may serve as a guide for other evaluations of harm reduction policies internationally.

## Background

### Drug Policy Reform: decriminalization to reduce drug-related health harms

Policymakers are increasingly acknowledging the harmful consequences of drug prohibition [1]. Punitive drug policies have largely failed to reduce the prevalence of drug abuse [2], fuelled epidemics of HIV and hepatitis C virus (HCV) infection [3-6], and discouraged the implementation and use of needle/syringe exchange programs, opioid substitution treatment (e.g., methadone maintenance), and other evidence-based public health interventions [7,8]. Law enforcement and policing activities that typically accompany drug control initiatives also result in the criminalization and stigmatization of drug users [9,10], high incarceration rates [11,12], human rights abuses [13,14], and escalations in drug-related violence and corruption leading to considerable political instability [15]. Incarceration of drug users has also promoted HIV and tuberculosis (TB) coinfection in many resource-poor settings [10]. Amidst accumulating evidence that the “war on drugs” has failed to curtail drug consumption and trafficking while producing such unintended consequences, experts have called for structural interventions including drug policy reforms [16,17].

Liberalization of punitive drug policies represents one alternative model to reducing the social and public health harms associated with drug abuse [18]. In particular, decriminalization of drug possession is gaining popularity [19] and has been adopted by countries in Europe (e.g., the Netherlands, Switzerland, and Portugal) [20] and the Americas (e.g., Argentina, Bolivia, Brazil, Columbia, Mexico, Peru, and Venezuela) [21-23]. Although no national drug policy reforms have been formally evaluated to date, Portugal’s 2001 decriminalization is arguably the best studied. Portugal’s reforms, which changed possession from a criminal to administrative offense and increased public health resources for drug abuse treatment and prevention, have been considered successful in reducing drug-related deaths, preventing transmission of HIV and HCV, and increasing utilization of syringe exchange programs and drug treatment services including methadone [24,25]. However, additional research is needed in contexts outside of Portugal’s relatively stable drug markets, functional police governance, and availability of high quality, evidence-based drug treatment services.

#### *Reforms in Mexico*

Recognizing the unintended consequences of punitive drug policies including dramatic increases in violent crime [16,17,19], Mexico recently enacted federal reforms involving decriminalization and expansion of treatment services [26]. This legislation, passed in 2010, decriminalized possession of small, specified amounts of drugs for personal use, including heroin (≤50 mg), cocaine (≤0.5 g), methamphetamine (≤40 mg), and marijuana (≤5 g) [27]. The law specified that, in lieu of arrest, persons apprehended with lesser amounts of these drugs would be released with only a police record until their third apprehension, when they would be required to either enter drug treatment or be incarcerated for 90 days. At the same time, Mexico developed plans for national expansion of drug treatment services. Legislation has been enacted by states using varying strategies and timelines.

Proponents of Mexico’s reforms have argued that law enforcement efforts will be appropriately redirected toward curtailing drug trafficking, allowing public health resources to be dedicated to new drug treatment services for drug users [26]. However, opponents have raised concerns about unintended consequences (e.g., increasing drug availability, overdose, drug trafficking, or “drug tourism” from countries with stricter drug laws) and because, unlike Portugal, evidence-based drug treatment options remain scarce in Mexico. Nevertheless, public funding to expand drug treatment and methadone services is increasing, suggesting the possibility of growing numbers of drug users beginning drug treatment throughout Mexico over the coming years.

### Epidemiology of HIV, HCV, and TB in the U.S.-Mexico Border Region

Understanding the impact of the recent reforms on the health of drug users is of great interest to public health officials in Mexico, where the HIV epidemic likely stemmed from the U.S. epidemic. The first three cases of HIV in Mexico were all identified in individuals who had visited the United States [28]. The early epidemic was concentrated among men who had sex with men (MSM) [29]. Blood donation in the early 1990s likely increased the spread of HIV to other subgroups [30]. Although HIV prevalence remains low in Mexico overall (overall prevalence was estimated at 0.3% in 2009), transmission has been closely linked to drug abuse, particularly in the Northern border region [31].

In cities such as Tijuana, Baja California, drug-related HIV risk behaviors are of particular concern. “Spillover” from heroin, cocaine and methamphetamine trafficking routes destined for the United States has contributed to increasing prevalence of drug abuse, especially injection drug use [32,33]. Along the West coast of Mexico, the lower purity “black tar” heroin remains more common than the powder forms, causing heroin users in this region to transition from smoking to injection more quickly [34]. In 2008, 4.8% of the population of Baja California reported injecting drugs, compared to 0.2% in Mexico overall [35]. Baja California also had the highest prevalence of methamphetamine abuse. Tijuana, where HIV has been associated with drug abuse, arguably has the largest number of people who inject drugs (PWID) per capita of any city in Mexico [29].

Baja California has the second highest cumulative AIDS incidence in Mexico (following the Federal District), comprising 4.7% of all AIDS cases in the country [31]. In 2006, an estimated one in every 116 persons aged 15–49 years in Tijuana was infected with HIV [36]. However, transmission, which has also been associated with active syphilis, remains concentrated among high risk populations including PWID (HIV prevalence of 4% overall; 3.5% among men and 10.2% among women) [29] and female sex workers ([FSWs]; HIV prevalence of 6%) [37]. Consistent with studies worldwide showing that PWID are at heightened risk for HCV infection from sharing syringes and injection paraphernalia, PWID in Tijuana have an extremely high HCV prevalence at 95% [38,39]. TB incidence among PWID in Tijuana is also high at 34 cases per 100 person-years [40], suggesting prevalent untreated, active infection. The majority of PWID (67%) and non-injecting drug users (58%) in Tijuana have been shown to have latent TB infection (LTBI) [41], and their risk of progressing to active TB increases from 10% over their lifetime to 10% annually if they are coinfected with HIV [42].

#### *Injection drug use in Tijuana, Mexico*

We have been conducting epidemiologic and behavioral surveillance among PWID in Northwestern Mexico’s border region for a decade. *Proyecto El Cuete* (“*cuete*” is slang for needle/syringe) represents a series of research studies that has qualitatively and quantitatively described injection behaviors [43,44], risk environments [45], and drug treatment experiences among PWID in Tijuana [46]. Although 95% of enrolled PWID reported injecting heroin (alone or in combination with cocaine or methamphetamine), more women than men used methamphetamine (80% vs. 68%, p < 0.01) [47], a concerning finding due to the lack of effective treatment strategies for methamphetamine abuse.

The *El Cuete* studies also helped link drug-related harms among PWID to characteristics of the broader social and policy environments. As in other settings [6,48-51], police practices in Tijuana have exerted powerful influences on risk behaviors among PWID [44]. In particular, findings revealed that unsanctioned arrests for having “track marks” (evidence of injection) and confiscation of syringes were commonly experienced by PWID and were associated with higher prevalence of HIV infection and associated injection-related risk behaviors [52-54]. Specific behaviors associated with policing practices include higher frequency of injection [55], receptive syringe sharing [56], seeking injection assistance from others [57], and injecting in public places or shooting galleries [52,56]. These findings underscore the importance of law enforcement and the legal environment in which blood borne infections are transmitted. However, the extent to which Mexico’s drug policy reforms could positively influence policing practices while reducing incarcerations and improving injection contexts for PWID in Tijuana remain unclear.

It is also unclear how quickly Baja California and other states in Mexico with a heavy burden of drug abuse can scale up evidence-based harm reduction services. While syringe exchange programs are rare in Mexico, syringes can be legally purchased without a prescription in pharmacies, which is the only way for many PWID to obtain sterile syringes. An observational study of PWID in Tijuana found that 81% reported purchasing syringes at pharmacies. However, within this subgroup, 16% reported being refused syringes or overcharged, which was associated with receptive syringe sharing, using syringes multiple times (e.g., more than five uses per syringe), and having greater numbers of abscesses [58]. In qualitative interviews, PWID reported experiencing stigma and other important social barriers to successfully purchasing syringes at pharmacies, despite the fact that it is legal for them to do so [59]. In other settings, PWID who were unable to exchange or purchase syringes also reported sharing used syringes [60] and obtaining syringes from shooting galleries and other unsafe syringe sources [49]. While Mexico’s reforms may promote syringe availability, the risk of facing a third apprehension for drug possession while attempting to purchase syringes legally could lead PWID to obtain syringes from more discreet and higher risk sources.

Mexico’s reforms also call for a dramatic expansion of drug treatment services. Among Tijuana PWID who reported ever receiving help with drug cessation in a 2004–2005 survey, most (80%) attended residential programs based on the “*ayuda mutua*” or twelve-step approach [46], despite the lack of evidence regarding the efficacy of such programs in Mexico. In a qualitative subsample of these PWID, nearly one quarter of those who had ever attended residential treatment programs described being physically or verbally mistreated by treatment center staff [46]. Some even reported preferring incarceration to drug treatment due to the negative reputations of rehabilitation centers [46]. At the same time, the observed low uptake of methadone maintenance in Tijuana has been attributed to the high cost (approximately $10 /day), scarcity of services, negative experiences with dosing, and perceptions of ineffectiveness [61]. However, some U.S. residents have reported preferring methadone treatment in Mexico [61]. While Mexico’s new drug policy includes funding that will increase treatment availability and encourage drug users to attend drug treatment rather than incarceration, the quality and effectiveness of programs will require close evaluation.

#### *Injection drug use in San Diego, United States*

The potential impact of Mexico’s policy reforms on the health and wellbeing of drug users in the United States also remains understudied. Tijuana and San Diego share the world’s busiest land border crossing, and their combined population of nearly six million residents is the largest of all metropolitan regions along the 2000 mile-long border [62]. The U.S.-Mexico border region is also characterized by some of the most dramatic income disparities between two adjacent countries in the world [63]. Due to the dearth of published literature on PWID in San Diego, despite recently published data from Tijuana, epidemiologic and behavioral surveillance of PWID in San Diego was started in 2009.

The “Study To Assess Hepatitis C Risk” (STAHR) was a cross-sectional study of HIV and HCV infection among PWID recruited in San Diego using street outreach, respondent driven sampling (RDS), and venue based sampling [64]. Although STAHR revealed that HIV prevalence (4%) among PWID in San Diego was comparable to that among PWID in Tijuana (4%) [53], it also pointed to vast disparities between the two cities in the prevalence of HCV and TB. As noted above, nearly all PWID in Tijuana (95%) are HCV-positive [38,39], while PWID in San Diego were found to have comparatively low HCV prevalence (27%) [64]. Similarly, in contrast to the high prevalence of LTBI among PWID in Tijuana [65], less than 15% of HIV-positive PWID tested in a San Diego HIV clinic were found to have LTBI [40,41]. The disparities in disease prevalence between Tijuana and San Diego raised concerns that PWID travelling from the United States to Mexico to buy or use drugs could face increased risk of acquiring these diseases through social, sexual, or drug use interactions [64].

### Cross-Border HIV Transmission: rationale for drug tourism research

Research in other settings has documented how dual epidemics of heroin injection and HIV follow drug trafficking routes across international borders [66]. A study of PWID in the China-Vietnam border region [67] identified a “gradient” of HIV prevalence that increased as heroin originating in Southeast Asia moved north (i.e., from northern Vietnam to southern China) [68]. Studies have also identified extensive international “drug tourism” from countries with restrictive drug laws governing the sale, possession, and use of drugs, to countries with more liberal policies [69,70]. Many U.S. residents travel to Mexico and Canada to obtain inexpensive prescription medications [71-73]. PWID in San Diego have described initially being influenced to travel to Mexico due to the reputation of Tijuana as a party city with fewer restrictions on drinking [61]. For some, this early travel evolved to include illicit drug use; however, data on the prevalence of tourism for illicit drugs of abuse and the associated risks remain sparse. In contrast to the situation in Mexico, possessing any amount of illicit drugs is illegal in the United States. Due to lower perceived risk of penalties following Mexico’s drug policy reforms, these legal changes could create an atmosphere in which drug users residing in the United States initiate or intensify travel to Mexico to purchase or use drugs. Such drug tourism by PWID from San Diego to Tijuana could influence cross-border transmission of infectious diseases. In particular, individuals taking part in this type of tourism who acquire a blood borne infection could “bridge” these diseases into geographically separated social networks.

Preliminary cross-sectional data from the STAHR study indicated that two-thirds of participants had crossed the border from San Diego into Tijuana, and more than a quarter (27%) of this group had injected drugs in Mexico [64]. Injecting in Mexico was associated with injecting heroin, distributive syringe sharing at least half of the time, and transporting drugs [74]. In qualitative interviews, PWID who injected in Mexico reported generally heavier drug use and greater familiarity with the border region [61]. Through qualitative interviews, they described the drug market in Tijuana as more visible and perceived that drugs were more readily available in Tijuana. Travel to Tijuana served as an option for some PWID to procure drugs when they were unable to find them in San Diego. While PWID from Tijuana were less likely to travel into San Diego due to U.S. customs restrictions, which generally require a passport, and limited mobility (particularly due to U.S. deportation records) [75], nearly half (48%) of PWID in Tijuana reported ever injecting drugs with someone from the United States [76].

This preliminary work left two key questions unanswered. First, while it was evident that a substantial proportion of PWID in both countries had crossed the border to use drugs, little was known about the social context of this drug use. For example, it was unknown whether PWID who travel from San Diego to Tijuana to purchase and use drugs were injecting with fellow travelers from San Diego or with PWID who reside in Mexico. Second, given the rapidly changing nature of policy reforms, including both Mexico’s policy reform and changing U.S. border enforcement policies, we expected that patterns of, and motivations for, drug-related border crossing would evolve over time. Given the existing patterns of drug tourism, it appeared possible that Mexico’s reforms could inadvertently increase cross-border travel for drug use, carrying with it multiple drug-related adverse effects on health.

Based on the need to assess the effects of Mexico’s drug policy reforms on drug abuse and related harms in the U.S.-Mexico border region, we developed a binational research agenda involving two parallel studies: a fourth phase of *Proyecto El Cuete* in Tijuana (i.e., *El Cuete IV*) and a second phase of the STAHR study in San Diego (i.e., STAHR II). Designed and implemented in tandem, these two studies provide the opportunity to evaluate the consequences of one country’s drug policies on the behaviors and wellbeing of drug users in two neighboring countries. The overall objective of *El Cuete IV*, planned for 2010–2015 but extended through 2020, is to assess the impact of Mexican drug policy reforms on HIV-related risk and protective factors among PWID in Tijuana, Mexico. Similarly, the objective of STAHR II, planned for 2010–2015, is to assess the impact of Mexico’s drug policy reforms on the knowledge, behaviors and infectious disease status of PWID in San Diego, CA. Specific aims of these projects include evaluating the effects of Mexico’s reform on knowledge, attitudes, risk and protective behaviors, experiences with incarceration and drug treatment, and prevalence and incidence of overdose, HIV and other infections, and death among PWID in Tijuana and San Diego.

#### *Conceptual framework*

To guide this binational research agenda, we developed a multilevel framework with two primary conceptual underpinnings. First, we drew from Rhodes’ Risk Environment Framework [10,77] to highlight the multilevel, contextualized nature of HIV risks and better hypothesize relationships between macro- and micro-level factors. Rather than solely emphasizing individual-level risk factors, this framework allowed us to emphasize how macro-level structural changes (e.g., national drug policy reforms) could influence health outcomes. Second, we drew from the Diffusion of Innovations Theory [78] to understand the processes by which information about the new policies and associated attitudes spread through social networks over time [79]. This framework has been tailored to the unique research questions and aims of each study and serves as a guide for our iterative mixed-methods approach detailed in the next sections of this paper.

## Methods

### Overview of binational mixed methods studies

*El Cuete IV* and STAHR II are prospective, mixed-methods observational cohort studies that were designed in parallel to allow triangulation of data from both cities. Between 2012 and 2014, we recruited separate, non-overlapping cohorts of PWID in each city to undergo semi-annual behavioral and biological assessments. Based on extensive binational collaboration and research experience with PWID in this region, U.S. and Mexican investigators interacted regularly to develop and refine complementary protocols and implement the two protocols in tandem. *El Cuete IV* enrolled 785 PWID in Tijuana to undergo behavioral assessments and HIV testing at baseline and every six months for 24 months, while STAHR II enrolled 575 PWID in San Diego to undergo behavioral and biological assessments for HIV, HCV and *Mtb* testing at baseline and every six months for 24 months. Although these studies were designed to be similar, we anticipated important differences in each context and developed mixed methods designs that could be responsive to the needs of each study site. Thus, subsamples of participants in both studies were selected after behavioral assessment visits to complete qualitative interviews to enhance our understanding of emergent phenomena and the diverse contextual factors surrounding our research aims. All procedures within both studies were approved by the Human Subjects Protections Program of the University of California, San Diego.

### Study populations

#### *Sampling and recruitment*

In both studies, sampling was driven by quantitative and qualitative study aims. We restricted sampling to PWID because they represented a group of drug users at elevated risk for blood-borne infections and tend to be more heavily affected by changes in the drug scene (e.g., new drug formulations that could change as a result from the drug policy reforms). Rather than continuing to follow *El Cuete IV* participants from the previous phases of our binational research agenda, we decided to recruit different individuals due to attrition, mortality, and other temporal trends that affected our previous cohorts.

We sought to obtain representative samples of PWID in each site. Although we initially considered recruiting new cohorts of PWID using respondent driven sampling (RDS), due to issues of cost and the limited effectiveness of RDS at recruiting female PWID [38,54], we instead used targeted sampling consisting of street-based outreach in diverse geographic areas [80]. For example, *El Cuete IV* outreach teams established temporary mobile recruitment sites (e.g., vans and tents) in ten distinct *colonias* (neighborhoods) characterized by different physical risk environments and where PWID were known to spend time. Once situated in these neighborhoods, outreach workers attempted to engage individuals in conversation, sometimes by offering HIV prevention materials or information (e.g., condoms, educational pamphlets). Due to the drug related violence throughout Mexico, we developed an approved safety protocol in which outreach workers conducted all field-based recruitment activities in pairs or teams rather than alone and always carried identification cards to provide to potential participants and authorities. While all outreach workers in Tijuana spoke Spanish fluently, we also included at least one English speaker in each outreach team given the high numbers of U.S. deportees in our target population.

STAHR II also used targeted sampling methods [80]. Recruitment involved direct street- and venue-based outreach (e.g., outreach workers passed out recruitment cards at parks, beaches, local syringe exchange vans and other areas where PWID congregate); targeted advertising through local newspapers, websites (e.g., Facebook, Craigslist), and posting flyers in neighborhoods and venues with a high concentration of PWID; and social networking strategies (e.g., peer referrals) within the target population. In addition, the study developed a website (http://www.ucsd-stahr.com) for potential participants to access information about the study. All written materials were available in English and Spanish. The outreach coordinator also maintained close contact with health centers and community agencies serving PWID (e.g., local syringe exchange programs). To better reach areas outside of the downtown area where STAHR II maintained a primary study office, a mobile outreach van and temporary office spaces were used to recruit PWID in northern, eastern, and southern San Diego County communities. These mobile sites were equipped to conduct all of the screening and data collection procedures described below. The STAHR II outreach staff also worked in pairs or teams, received study related trainings, and followed specific safety precautions described in an approved injury and illness prevention plan.

#### *Eligibility criteria*

In both studies, eligibility criteria included the following characteristics: 1) being at least 18 years of age, 2) having evidence of injecting illicit drugs within the past month (i.e., confirmed by observation of track marks or other physical evidence of injecting), 3) being able to converse in English or Spanish, 4) currently residing in the study city with no plans to move away within 24 months from enrollment date, and 5) not currently participating in any intervention studies (although none to our knowledge were being conducted). Individuals with severe cognitive deficiencies or who were unwilling to provide informed consent were excluded, and PWID who met eligibility criteria but were too intoxicated to provide informed consent were rescheduled for rescreening at a later date.

#### *Screening*

Eligibility screening for both studies was conducted in private rooms in primary study offices (both located in commercial buildings) or at alternate sites (e.g., an offsite clinic, mobile van, or tent set up in outside venues). Screening began with the provision of general information about the study aims and procedures. Potential participants were asked for verbal consent before beginning the screening interview. Screening instruments were interviewer-administered, lasted approximately five minutes, and included several extraneous questions designed to prevent potential subjects from guessing eligibility criteria. Individuals were reimbursed $5 USD for their time to complete the screening, regardless of eligibility. Ineligible individuals were also offered free condoms, information and referrals for HIV testing, and reimbursement for public transportation as appropriate.

#### *Informed consent*

Following screening procedures, eligible PWID were asked to provide written informed consent. Interviewers handed potential subjects a copy of the consent form, read through the consent document to highlight key content, and discussed the study risks and benefits with the subject to determine understanding of the procedures and answer any questions that subjects had. In both sites, these materials were available in English and Spanish. Eligible individuals who decided to participate were asked to sign the consent form and were then invited to immediately begin baseline data collection unless they preferred to be rescheduled for a later time, which could require another eligibility screening process to ensure that time-dependent criteria were still met.

#### *Qualitative subsamples*

Subsamples of participants in each study were invited to undergo in-depth interviews to obtain qualitative data. Participants were purposively sampled on certain characteristics (captured in quantitative described below) to explore knowledge and behaviors in the context of Mexico’s legal reforms. Approximate sample sizes were determined based on the principle of conceptual saturation [81], which occurs when conducting additional interviews fails to provide major new findings [82]. Due to the complexity of themes that we intended to explore, and the possibility of interviewing new participants and considering additional emergent themes during subsequent waves of qualitative data collection, we decided to initially conduct qualitative interviews with approximately 20 PWID per wave. During qualitative data collection, investigators held regular meetings in which interviewers presented summaries of new and recurring findings so that the research team could together evaluate saturation. Our mixed methods designs were sufficiently flexible to allow conducting qualitative interviews with additional participants when new themes were identified and required more in-depth exploration. Since the qualitative samples were fully embedded within the longitudinal cohorts, contacting additional participants was facilitated by a robust tracking system designed for retention of participants over multiple visits, as described below.

#### *Retention and follow-up considerations*

We developed active and passive follow-up strategies based on our binational team’s successful experience following PWID and other marginalized populations. During enrollment for both studies, staff members collected locator data on separate forms to facilitate participant follow-up. For example, locator forms collected information on where participants lived and spent time, their direct contact information (if available), individual physical characteristics, and family/social contacts of participants. Mailing addresses were unavailable for many of our participants, so one tracking strategy utilized in *El Cuete IV* involved asking participants to locate on maps where they lived (e.g., vacant lots, parks, canyons, shelters) and spent time during different hours of the day (e.g., panhandling spots, bars, clubs, food establishments). Participants also received small, wallet-sized appointment cards at enrollment that contained reminders of study visits, incentive schedules, locations and contact information for the study offices.

From our past cohort studies, we anticipated that repeated contact with participants would improve retention, so *El Cuete IV* and STAHR II compensated participants with $5 USD for brief “check-ins” at intervals halfway between semi-annual assessment visits (e.g., at months 3, 9, 15, etc.). Outreach workers conducted check-ins in person or by telephone, which involved asking participants to update their locator information and reminding them about future study appointments. All locator data were maintained in a password protected locator database stored separately from interview data to ensure confidentiality. Each month, data managers provided staff with calendars and lists of participants who were due for primary and locator visits. Both studies developed logos that were used throughout all methods of recruitment and tracking to enhance participants’ recognition and recollection of the study.

Staff also engaged in office- and street-based tracking of participants. Office-based tracking involved phone, text, email, postcard, and birthday card reminders, as well as phone calls to family/social contacts listed on locator forms. After three unsuccessful contact attempts, outreach workers proceeded to conduct home visits and other forms of street-based tracking (e.g., visiting locations where participants indicated that they spent time). Outreach staff also posted general notices describing the study in English and Spanish at shelters, bus terminals, airports, syringe exchange sites, health clinics, and drug treatment programs on both sides of the border. Due to the high numbers of migrants deported from the United States to Tijuana each year, STAHR II utilized administrative data to help locate participants who were incarcerated in or deported to Mexico. At the same time, *El Cuete IV* periodically searched for participants in neighborhoods where they were recruited, homeless day centers (i.e., *desayunadores*), and prisons and drug treatment centers.

Other retention strategies were specific to the contexts of each site. Based on our research experience with mobile PWID in San Diego, many of whom were unstably housed, STAHR II also utilized escalating monetary reimbursements and small gifts (e.g., toiletries, condoms, ID holders) to promote retention. For example, STAHR II reimbursed participants $25 for completing baseline surveys and testing, $25 at their 6-month visit, $30 at their 12- and 18-month visits, and $50 for their final, 24-month visit. In between these data collection visits, participants could also receive $10 for returning to receive their biological test results three weeks after each assessment visit, and additional $5 payments for completing the locator visits described above. Participants were informed that the total reimbursement amount they could receive over the two-year course of the study was $235. STAHR II also maintained the study website (http://www.ucsd-stahr.com) and accounts on popular social networking sites (e.g., Facebook) to keep in touch with participants. Finally, STAHR II gave participants wallet-sized calendars with toll-free phone number accessible from the United States and Mexico. Despite the unique contexts of the lives of PWID in both settings, both studies (and the experienced staff members dedicated to each project) were able to inform the other with respect to retention and tracking strategies.

### Data collection

Data collection strategies were intended to be similar across both studies, but we also recognized how studying this unique historical, binational situation would benefit from study designs that were flexible and responsive to local events and changes in risk environments over time. Both studies collected quantitative (survey) data and biological samples from the overall cohorts at baseline and semi-annual follow-up visits. Embedded subsamples of PWID were also selected from the two cohorts based on each study’s specific aims to complete qualitative interviews at multiple time points [83]. Careful monitoring of all forms of data collection was inherent in our study designs and allowed for quantitative and qualitative instruments to be refined in-between study visits to capture new, emergent phenomena.

#### *Quantitative data collection*

For both studies, quantitative instruments were administered in English or Spanish by trained bilingual interviewers in confidential settings. Assessment instruments for both studies were developed jointly by the same team of investigators with the intention of creating identical measures whenever possible and to provide information from one study to complement data from the other study. For example, the *El Cuete IV* assessed experiences engaging in drug use with U.S. PWID to provide context for questions in the STAHR II assessment about injecting behaviors while in Mexico. This collaboration in measurement was intended to facilitate joint analyses including data from both cohorts. Bilingual, bicultural study staff members who were familiar with the unique language of the border region translated instruments from English into Spanish as necessary. The bilingual project director then back-translated these instruments into English to assess accuracy. Bilingual interviewers administered surveys using computer-assisted participant interview (CAPI) technology, which we have used for previous studies in Mexico and the United States.

Quantitative instruments at baseline assessed lifetime and recent experiences and behaviors, while follow-up surveys emphasized the time elapsed since the prior interview (six months). Socio-demographics measures included race/ethnicity, place of birth, education, language proficiency, citizenship and immigration status, passport ownership, marital status, living situation, binational travel, migration and deportation experiences. Measures of knowledge and attitudes focused on Mexico’s recent drug law reform, the health risks of using/injecting drugs following reforms, and, for STAHR II participants, travelling to Mexico to use drugs. For example, due to recent drug-related deaths in Mexico and shifts in methamphetamine production from California to Baja California [32,33], knowledge and perceptions among PWID in San Diego regarding drug manufacturing could influence their perceptions about the risks and benefits of using drugs in Mexico.

Drug use behaviors included lifetime and recent use of specific drugs and routes of drug administration (e.g., sniffing, smoking, swallowing, injecting), including routes that could increase TB exposure (e.g., sharing pipes and “shot-gunning,” the exhalation of smoke directly into another person’s mouth) [84,85]. We also assessed syringe and drug acquisition and periodically added new items in response to reports about emerging trends were worth monitoring in high risk communities (e.g., synthetic drugs such as “bath salts” [i.e., synthetic cathinones]). Drug treatment measures included lifetime and recent experiences with voluntary and court-mandated drug treatment involving diverse modalities (e.g. methadone, outpatient vs. residential drug treatment, self-help groups), barriers to accessing treatment, and perceptions regarding service quality/efficacy. Sexual risk behaviors for HIV transmission included number and types of partners, exchanging sex for money or other material goods, condom use, and drug use with sex partners. Other health measures included access to healthcare, history of prior diagnosis and treatment for active TB or LTBI and HCV, treatment type and completion, and symptoms [86]. Location data for key outcomes of interest (e.g., interactions with police) were obtained by showing participants electronic maps on laptop computers and asking them to indicate exactly where such events occurred.

#### *Serologic counseling and testing*

Both studies conducted serologic testing for HIV infection at each visit. Participants received pre- and post-test counseling according to the Mexican Ministry of Health (*El Cuete IV* only) and U.S. Centers for Disease Control (CDC) guidelines (both studies). Serologic testing involved blood specimens collected via fingerstick and venipuncture according to standard clinical practice by a trained phlebotomists who were experienced in obtaining blood from PWID with scarred veins. *El Cuete IV* used Advance Quality rapid HIV tests (InTec Products, Inc). Reactive rapid tests were repeated. Participants receiving a second reactive rapid test were considered positive and referred to nearby municipal health clinics for free care under Mexico’s universal health system (e.g., Centro de Salud No. 1 or CAPASITS).

In STAHR II, HIV testing was performed using Uni-Gold TM Recombigen® HIV rapid test on whole blood collected via finger stick. For reactive tests, confirmatory testing was performed using the OraQuick ADVANCE® Rapid HIV-1/2 Antibody Test (OraSure Technologies, Bethlehem, Pennsylvania, USA). For reactive, inconclusive, or discordant test results, additional blood samples were collected and sent for confirmatory testing at the San Diego County Public Health Laboratory. In addition to serologic testing, we collected whole blood specimens for HIV nucleic acid analysis using DNAgard® Blood Tubes (Biomatrica, San Diego, California, USA), which contain a stabilizing agent allowing blood to be stored at room temperature without sacrificing DNA integrity or recovery. Plasma and serum samples were also collected at each visit for storage for future studies.

STAHR II also tested for HCV and *Mycobacterium tuberculosis* (*Mtb*) infection. HCV testing was conducted using the OraQuick® HCV Rapid Antibody Test (OraSure Technologies, Bethlehem, Pennsylvania, USA). Given the accuracy of this test, no confirmatory HCV testing was performed. Participants testing HCV-positive were referred to their health care provider or assisted in identifying a provider who assess liver function and test for active versus resolve infection. Testing for *Mtb* infection was performed using the QuantiFERON® TB Gold In-Tube assay ([QFT] Cellestis, Carnegie, Victoria, Australia). QFT requires blood samples to be incubated overnight followed by enzyme-linked immunosorbant assay; thus, participants were invited to return to receive these results. Participants testing positive for *Mtb* infection required evaluation to rule out active TB and potentially receive treatment for LTBI. In these situations, counselors assisted them in making appointments with their own physicians or referred them to the San Diego County TB Control Program. In both studies, participants were retested for infections at each visit until receiving a positive result, after which they were no longer retested for that infection. Referrals and educational resources were also provided for substance abuse treatment, management of wounds and abscesses, liver care, domestic violence, hunger, and housing.

#### *Qualitative data collection*

In both studies, qualitative interview guides contained broad, open-ended questions addressing study aims could be amended to more specifically address emergent findings. Interview guides were intended to be unstructured enough to allow interviewers to explore these emergent issues throughout the interview. Interviewers were also extensively trained in conducting interviews in a nonjudgmental, conversational manner and probing for additional details relating to study aims. Qualitative interviews were digitally recorded and transcribed for content analysis. During and after each interview, interviewers wrote detailed summary notes on new and important findings and reflected on emergent themes across interviews to inform preliminary analyses. These notes were later presented during regular team meetings so that the research team could identify commonalities across interviews and discuss progress towards achieving conceptual saturation. Interviewers also used feedback forms that were specifically designed for each study to record additional information on interview quality, participant demeanor, nonverbal behavior or emotions, and their general thoughts following the interview. Interview transcripts, interviewer notes, and feedback forms were translated as necessary for biweekly phone and in-person team meetings and preliminary analyses.

#### *Other data collection*

The *El Cuete IV* study obtained permission to access administrative records from Mexican authorities at the municipal and state levels on the following outcomes of interest: 1) drug treatment programs (e.g., type of treatment, date of entry and exit, voluntary vs. court-mandated, methadone dose, etc.), 2) interactions with law enforcement (e.g., dates and types of arrests, numbers of strikes, released vs. referred to drug treatment, etc.), 3) incarceration (e.g., nature of conviction, date of entry and exit, etc.). We also searched public registries semiannually to obtain death certificates (e.g., using the Mexican *Registro Civil* or *SEMEFO*). STAHR II also obtained access to publically available incarceration and reviewed death records to determine the status of participants who were lost to follow-up.

### Data analysis

Overall data analysis procedures were intended to be similar across our two mixed methods studies while also permitting flexibility given the different settings and unique study aims.

#### *Quantitative data analyses*

For both studies, descriptive statistics provided summary measures to describe initial and changes in knowledge, attitudes, experiences and behaviors in relation to Mexico’s reforms (e.g., means, standard deviations, medians, interquartile ranges, proportions). Frequency tables, bar charts, and line graphs helped visualize the data. Bivariable, mediation and multivariable regression analyses assessed potential correlates of our dependent variables and identified potential interactions [87]. Longitudinal analyses (e.g., using generalized linear mixed models) were planned for determining the extent to which knowledge, attitudes, experiences and practices changed over time and in relation to the implementation of legal reforms. Geographic analyses of location-based data were also planned to help identify “hotspots” of key events and outcomes of interest.

#### *Phylogenetic analyses*

In addition to statistical analyses, whole blood specimens from HIV infected individuals underwent HIV nucleic acid analysis. Samples were processed to extract DNA and then amplify and sequence the *pol* region of HIV. These sequences were then compared to our existing research database of ≥1,000 HIV sequences from across the San Diego-Tijuana border region. Utilizing a network approach, we identified putative transmission linkages between sampled sequences by calculating the genetic distance between each pair of sequences (TN93 distance). We considered a linkage to be present when a pair of sequences was found to be <1.5% different, a conservative threshold based on our previous work in this area [88].

#### *Qualitative data analyses*

Occurring in tandem with data collection, qualitative analyses have followed a thematic approach [83], drawing from our conceptual framework to explore the multi-level, contextualized nature of HIV and drug-related harms in this border setting [10,77] and the ways in which knowledge and attitudes spread through social networks to affect health outcomes [78]. Interviewers and investigators attended regular meetings to discuss preliminary findings, develop coding schemes collaboratively [89], and identify emergent concepts to explore in future waves of data collection and analysis [90]. Supplemental analyses of emergent themes were also planned.

### Results & discussion

Through a detailed explanation of our methodological approach, we demonstrated the feasibility of implementing binational mixed methods studies that borrow from the principles of a “natural experiment” to evaluate the effects of a high level structural intervention (i.e., a national policy change) in two diverse but interdependent contexts. Through the unique collaboration represented by *El Cuete IV* and STAHR II, which involved extensive interaction between investigators and research staff across an international border, we successfully recruited two parallel cohorts of PWID in San Diego (United States) and Tijuana (Mexico). We believe that our experiences can help inform future research on the impact of drug policy reform on the health and wellbeing of drug users in other international settings.

Overall, our prospective, mixed methods study design was both exploratory (i.e., qualitative data helped develop and refine conceptual models for how drug policy reform impacts the behaviors and experiences of PWID) [91], and complementary (i.e., findings from different study components led to different but complementary contributions to our understanding of complex local phenomena) [92,93]. The iterative process of using qualitative and quantitative data to inform the methods and interpretations of results from each study component [92,93] contributed toward a more comprehensive understanding of the binational effects of Mexico’s drug policy reforms on the health and wellbeing of PWID. Our integration of multiple methods of inquiry generated important new hypotheses, refined analysis plans, and enhanced interpretations of preliminary findings. While traditional epidemiologic survey could miss the key aspects of local contexts, a small qualitative study could not necessarily capture larger trends in drug abuse behaviors across this international border. Utilizing multiple research methods allowed us to offset the weaknesses of each individual method and helped address questions that could not have been answered through single methods alone.

We integrating different research methods in several ways that have been recommended in the literature [94]. First, as our qualitative samples were embedded within two larger cohorts, we were able to use quantitative datasets to identify potential qualitative interviewees who met our purposive sampling criteria [83]. For example, we used quantitative data to identify STAHR II participants who lived in different geographic areas of San Diego County to compare their experiences traveling to and using drugs in Mexico. Second, quantitative data also provided strata on which qualitative themes could be compared. Third, while quantitative data allowed us to describe behaviors, geographic hotspots and associated outcomes, our qualitative data provided more rich, narrative context on the reasons, meanings, and processes underlying quantitative associations, leading to enhanced interpretations of quantitative results. Fourth, emergent qualitative findings led to the development of additional quantitative hypotheses. Finally, our utilization of HIV sequence analysis and newer phylogenetic and network based analytic tools enabled us build upon our quantitative findings regarding prevalence and incidence by providing objective evidence about cross-border population mixing and the implications for HIV epidemics on both sides of the border.

An essential feature of our research agenda was the flexibility built into our approach, which allowed preliminary findings from different study components to inform other study components (e.g., preliminary qualitative findings from STAHR II helped refine future waves of quantitative survey data collection in STAHR II and *El Cuete IV*) [91]. We believe that conducting a comprehensive evaluations of high level structural interventions such as national drug policy reforms demands a research design that can be responsive to local phenomena that evolve over time. By embedding a flexible, prospective qualitative component within two larger cohort studies, we were able to conduct real-time assessments of ongoing events while also understanding evolution in the perceptions of PWID living through those events.

In both studies, flexible sampling strategies have allowed the following-up on emergent themes during subsequent waves of data collection. For example, in *El Cuete IV*, we initially sought to conduct qualitative interviews with participants who had received varying numbers of drug possession apprehensions. Upon learning that implementation of the reforms had been severely delayed and few PWID in Tijuana had received any strikes at baseline, we modified our qualitative sampling criteria to include any participants who reported being stopped with and without being arrested. Our ability to proactively seek additional qualitative respondents meeting the new criteria was heightened by our use of quantitative survey data [83]. Flexibility has also afforded us the ability to follow-up on preliminary quantitative findings using subsequent rounds of “member checking” qualitative interviews that solicit participants’ feedback on findings and investigators’ interpretations [95].

This type of flexible and iterative sampling is a hallmark of traditional qualitative designs [96] that is rarely integrated into large epidemiologic cohort studies. We argue that there are multiple benefits of having a highly responsive qualitative component but also acknowledge that it requires additional inputs from investigators and staff [97]. In our case, to meet this challenge, we have held regular meetings to discuss emergent findings, inform investigators and staff working on the other parallel study, and revise quantitative instruments accordingly. Investigators and analysts also communicate across sites regularly to share and confirm preliminary findings across studies and identify emergent topics that warrant additional investigation. Although time-consuming, we agree with others that this iterative, flexible, and collaborative approach yields more inclusive and nuanced findings with important program and policy implications [98,99].

A final strength of our research agenda is our ability to train a diverse new generation of harm reduction researchers and practitioners by leveraging support from the National Institutes of Health and the Fogarty International Center. Examples of funding that we have obtained include career development grants (e.g., Mentored Research Scientist Development Awards [K01 grants]), institutional research training grants (e.g., T32 for pre- and postdoctoral trainees), and diversity-promoting fellowships and supplements. To date, the *El Cuete* series has trained over 50 graduate students, medical students, fellows and junior faculty from institutions on both side of the border including the two largest public universities in this region: the University of California, San Diego (UCSD) and the Universidad Autónoma de Baja California (in Tijuana). The STAHR series has utilized similar funding opportunities to train 16 students from the high school through postdoctoral levels, as well as medical residents and junior faculty from UCSD and San Diego State University. Eleven of these trainees belong to racial/ethnic minority groups. While the primary objective of our training agenda has been to provide interdisciplinary research opportunities for students and new investigators, particularly those from underrepresented minority groups in the sciences, including diverse perspectives and expertise within our team has also enhanced the quality and meaningfulness of our research.

In designing our binational protocol, we considered several potential limitations. First, our studies depend largely on highly sensitive, self-reported behaviors. However, there is an extensive literature finding drug users’ self-reported behaviors to be valid [100-102]. As an additional strategy for guarding against underreporting of risky behaviors, we consistently assure participants that all data is confidential and that we do not report any behaviors or identities to authorities. To provide U.S. participants with greater assurance that their data will be kept confidential, STAHR II obtained a Certificate of Confidentiality. To the extent possible, we attempt to confirm some behaviors and experiences using objective administrative data (e.g., from drug treatment programs, police records, and jail/prisons). We also provide staff members with extensive training to increase their familiarity with the drug scene (e.g., local drug abuse trends, slang currently used on the street), which we believe enhances the rapport that they are able to develop with participants. We believe that this training and the resulting ability of staff members to interact comfortably with participants in a nonjudgmental manner serves to improve the reliability of self-reported behaviors.

Other important limitations of our research relate to sampling. Due to our nonrandom sampling strategies, our results cannot be interpreted as representative of the entire populations of PWID in Tijuana or San Diego. Our data also have limited generalizability with respect to other populations of PWID internationally, especially in regions with different policies and where heroin and methamphetamine are not the major drugs of abuse. Sample size constraints may limit our ability to identify all hypothesized effects. However, to minimize attrition, our outreach teams were extensively trained in office- and street-based tracking and the use of multiple active and passive follow-up techniques. As part of our binational collaboration, we also share information about successful strategies between studies. We believe that our collaborative, mixed methods approach, combined with the innovation and fortuitous timing of our binational research, will offset many of these limitations.

## Conclusions

The description of our two parallel study protocols demonstrates the feasibility of implementing a binational research agenda designed to assess the impact of high level structural interventions on the health and wellbeing of PWID in Mexico and the United States. Our extensive collaboration, combined with our flexible, prospective mixed methods approaches, allowed us to address each study’s specific aims and document emerging phenomena within these diverse local contexts. By recognizing the complementary nature of qualitative and quantitative methods, our research strategy provided a more holistic and sophisticated understanding of the phenomenon under study than could be achieved by a single-method design. It is our hope that the description of these methods will help inform other research on the impact of drug policy reform on the harm reduction agenda.

## Abbreviations

CAPI: Computer-assisted participant interview; CDC: Centers for disease control and prevention; HCV: Hepatitis C virus; FSW: Female sex worker; LTBI: Latent tuberculosis infection; MSM: Men who have sex with men; PWID: People who inject drugs; QFT: QunatiFERON; RDS: Respondent driven sampling; STAHR: Study to assess hepatitis C risk; TB: Tuberculosis; UCSD: University of California, San Diego.

## Competing interests

The authors declare that they have no competing interests.

## Authors’ contributions

AMR wrote the initial draft of the manuscript; KDW helped design and field test the measures and processes; KDW, SRM, CMR, JCM and PGMZ contributed content and revised the manuscript; RSD and SAS conceived of the study designs, contributed content, revised, and provided final approval of the manuscript. All authors read and approved the final manuscript.
